# The appropriateness of low-acuity cases referred for emergency ambulance dispatch following ambulance service secondary telephone triage: A retrospective cohort study

**DOI:** 10.1371/journal.pone.0221158

**Published:** 2019-08-13

**Authors:** Kathryn Eastwood, Amee Morgans, Johannes Stoelwinder, Karen Smith

**Affiliations:** 1 Department of Epidemiology and Preventive Medicine, Monash University, Melbourne, Victoria, Australia; 2 Ambulance Victoria, Doncaster, Victoria, Australia; 3 Emergency Services Telecommunications Authority, Burwood, Victoria, Australia; Medical University Graz, AUSTRIA

## Abstract

**Objective:**

Ambulance-based secondary telephone triage systems have been established in ambulance services to divert low-acuity cases away from emergency ambulance dispatch. However, some low-acuity cases still receive an emergency ambulance dispatch following secondary triage. To date, no evidence exists identifying whether these cases required an emergency ambulance. The aim of this study was to investigate whether cases were appropriately referred for emergency ambulance dispatch following secondary telephone triage.

**Methods:**

A retrospective cohort analysis was conducted of cases referred for emergency ambulance dispatch in Melbourne, Australia following secondary telephone triage between September 2009 and June 2012. Appropriateness was measured by assessing the frequency of advanced life support (ALS) treatment by paramedics, and paramedic transport to hospital.

**Results:**

There were 23,696 cases included in this study. Overall, 54% of cases received paramedic treatment, which was similar to the state-wide rate for emergency ambulance cases (55.5%). All secondary telephone triage cases referred for emergency ambulance dispatch had transportation rates higher than all metropolitan emergency ambulance cases (82.2% versus 71.1%). Two-thirds of the cases that were transported were also treated by paramedics (66.5%), and 17.7% of cases were not transported to hospital by ambulance following paramedic assessment.

**Conclusions:**

Overall, the cases returned for emergency ambulance dispatch following secondary telephone triage were appropriate. Nevertheless, the paramedic treatment rates in particular indicate a considerable rate of overtriage requiring further investigation to optimize the efficacy of secondary telephone triage.

## Introduction

Some ambulance services estimate that up to half of patients calling for emergency medical assistance do not require an ambulance and could be better managed via alternative care pathways[1]. Patients with low-acuity conditions are increasingly calling emergency ambulances[2], forcing those requiring urgent medical attention to wait for resources responding from further away. In an effort to manage this increasing low-acuity demand[3] and the increasing costs of emergency ambulance response[4], some ambulance services have introduced secondary telephone triage to refer particular low-acuity cases to alternative care pathways[5–9]. This demand management strategy has managed up to 24% of the workload for some services[4, 10], however anywhere from 17%-71% of cases are referred back to emergency ambulance dispatch[6] and no evidence exists as to whether the decisions by triaging nurses or paramedics (clinicians) are appropriate.

Case-types selected for secondary triage often have historically low paramedic treatment and transport rates, and low re-presentation rates[2]. After calling the emergency telephone number cases that have no evident emergency symptoms and meet these criteria are referred for clinician-led secondary telephone triage. Ambulance Victoria, in Victoria, Australia, is supported by a range of non-emergency patient transport services not only capable of providing transportation, but also basic medical care[5]. In addition to this, home-visiting doctors, nurses and allied heathcare workers are contracted to Ambulance Victoria to respond to cases deemed suitable for these alternative care pathways following secondary telephone triage[5].

Currently, the paramedic treatment rate for all cases dispatched an emergency ambulance in Victoria is 55.5%[2]. It is unknown whether cases referred for an emergency ambulance dispatch following secondary telephone triage possess the same treatment rates (and therefore utilization rates of the paramedic skill-set) or whether their initial low-acuity triage was a better reflection of their potential need for an emergency ambulance dispatch. A study of the emergency department (ED) outcomes of cases referred to emergency ambulances following secondary triage found they were more ‘ED suitable’ and more likely receive a hospital admission than general cases arriving by emergency ambulance[7]. Conversely, cases triaged as inappropriate for emergency ambulance dispatch following secondary telephone triage were less likely to receive ED care or hospital admission if they subsequently presented in the ED[8, 9]. Despite this apparently ability to identify cases more suitable for the ED, no research has been conducted to determine whether these cases required an emergency ambulance prior to their ED presentation. The aim of this study was to investigate whether cases were appropriately referred for emergency ambulance dispatch following secondary telephone triage.

## Materials and methods

### Study design

A retrospective cohort analysis was conducted of secondary telephone triage cases referred for emergency ambulance dispatch between September 2009 and June 2012. Cases with a secondary telephone triage record and a corresponding paramedic patient care record were included in the study.

### Study setting

Ambulance Victoria is the sole provider of emergency medical services to the state of Victoria, Australia. Melbourne is the capital and largest city in Victoria, with a population of 4.25 million in 2012[11]. Between 2009 and 2012, Ambulance Victoria responded to just over a million cases in Melbourne using a two-tiered medical response system. The lower tier consists of Advanced Life Support (ALS) paramedics who are authorised to gain intravenous (IV) access, provide intravenous drug and fluid therapy, use a range of pharmacological agents, insert laryngeal mask airways, provide continuous positive airway pressure (CPAP) therapy, 12-lead electrocardiogram interpretation and perform chest decompression[12]. Mobile intensive care ambulance (MICA) paramedics form the upper tier and are authorised to perform additional procedures including rapid sequence intubation (RSI), cricothyroidotomy, elective cardioversion, mechanical ventilation, cardiac thrombolysis and administer a broader range of pharmacological agents. The decision to respond ALS paramedics, MICA paramedics or both is made using the Advanced Medical Priority Dispatch System (AMPDS) primary triage tool and an Ambulance Victoria formulated service allocation matrix[5].

**Fig 1:**
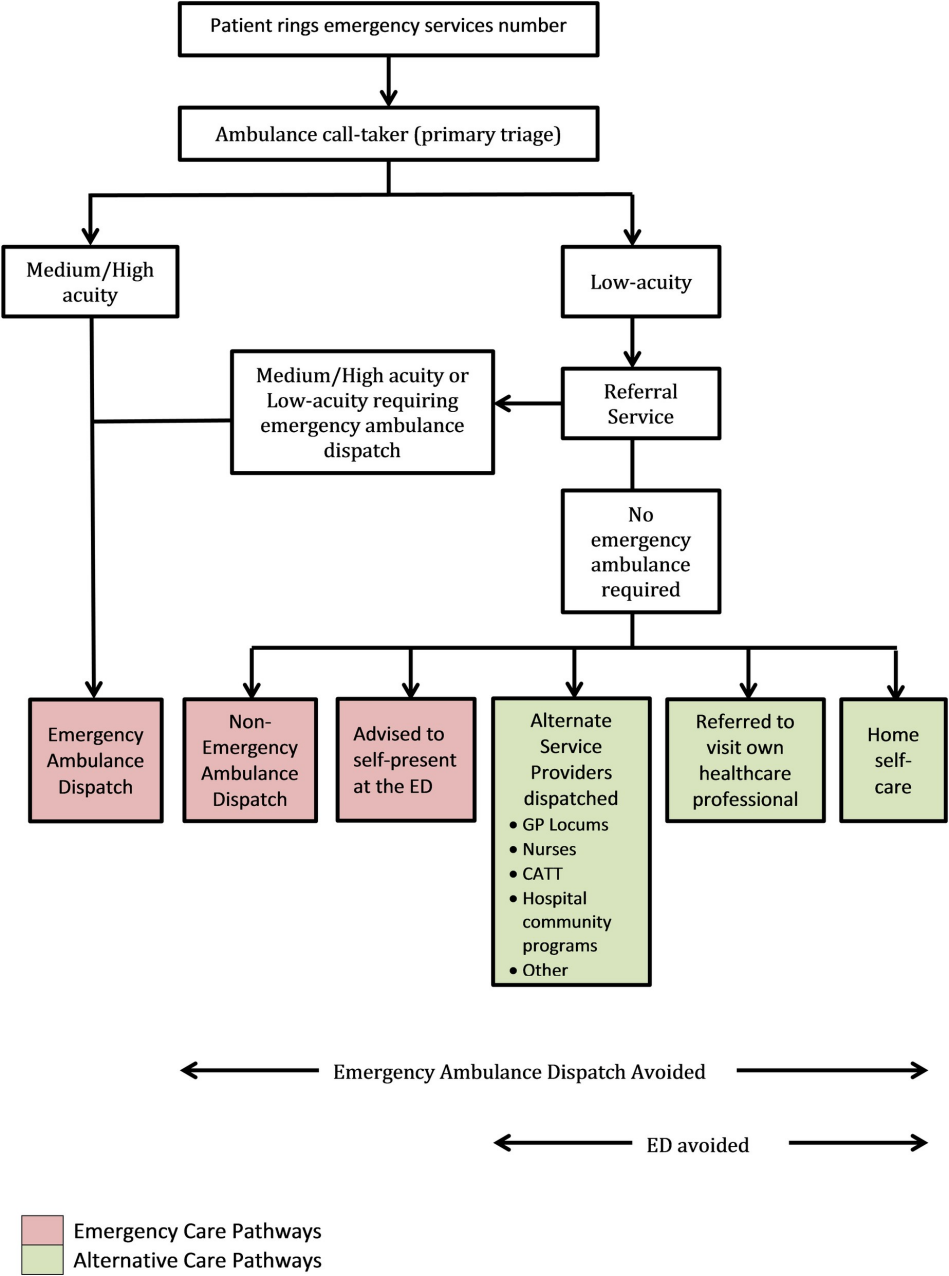
Referral Service care pathways.

The ‘Referral Service’ is a secondary telephone triage service operating 24 hours a day, seven days a week since 2003 and is a subsidiary of Ambulance Victoria. It provides secondary telephone triage to cases identified as low-acuity following primary triage. Qualified and experienced paramedics or nurses conduct the secondary telephone triage using a computer-based triage algorithm to ascertain the most appropriate disposition outcome for the case (Fig 1). The broad dispositions are:

- advice regarding the provision of self-care or to self-present at a community-based medical or health service or attend the ED;
- the dispatch of an alternative service provider including home-visiting doctors, nurses and hospital outreach programs;
- the dispatch of a non-emergency ambulance, or
- the dispatch of an emergency ambulance.

Non-emergency ambulance services operation state-wide in Victorian and are staffed by ambulance attendants qualified to administer basic life support (BLS) care. This includes oxygen administration, ECG monitoring and drug administration such as aspirin, glyceryl trinitrate, salbutamol, glucagon and non-opioid analgesia. They can also transport patients with established infusions including blood products. Non-emergency ambulance services are available 24 hours a day to receive appropriate cases from the emergency ambulance pathway.

Only the cases in the ‘emergency ambulance dispatch’ group that were referred following secondary telephone triage by the Referral Service were investigated in this study (Fig 1). The Referral Service has been described in more detail elsewhere[5].

### Data sources

Referral Service records were extracted from the Referral Service database and corresponding paramedic records for the cases referred for emergency ambulance dispatch were extracted and linked. These records documented patient demographic, assessment and treatment information, and operational information.

### Indicators of appropriateness

The appropriateness of triaging cases for emergency ambulance dispatch was analysed using paramedic treatment and paramedic transport as surrogate markers. A sub-analysis was conducted using dispatch acuity categories.

#### Paramedic treatment

Paramedic treatment has been used in this study, and others[3, 13–18], to suggest that cases that are not treated (i.e. do not receive ALS treatment) are more appropriate for transport or care pathways other than an emergency ambulance.

Treatment by emergency ambulance paramedics was defined as the utilization of ALS level treatment strategies with the intent of modifying the patient’s current physiological condition or medical presentation. This consisted of drug or fluid administration, oxygen therapy, perfusion or cardiovascular support management, and mental health management including chemical or physical restraint. Intravenous line insertion was excluded from ‘paramedic treatment’ if it was not subsequently used for drug or fluid resuscitation (ie. there was no intent to modify a patient’s physiological or medical presentation and therefore potentially not required)[15]. First aid, patient extrication, and tasks such as ongoing monitoring including glucose measurements, pulse oximetry and cardiac monitoring are within the non-emergency ambulance attendants scope of practice in Victoria and were therefore not included in the paramedic treatment definition. To ensure generalisability of the results to ambulance services where non-emergency transportation services are unable to provide any pharmacological intervention, any drug administration was considered an ALS treatment (ie. the drug administration identified as within the Victorian non-emergency ambulance attendants scope of practice was included as ALS treatment here).

#### Paramedic transport

Not all cases seen by paramedics are subsequently transported to the ED. Some cases are referred to self-present at the ED in a private vehicle. Others may be referred to community-based care or given definitive treatment on scene. In this study, paramedic non-transportation was used to identify cases potentially suitable for an alternative care pathway.

#### Dispatch acuity categories

The Referral Service call-takers use clinical judgment to decide upon the acuity of the patient and resultant urgency with which to respond an emergency ambulance when necessary. These acuity levels are:

- high-acuity–lights and sirens response within 15 minutes e.g. suspected cardiac arrests, severe respiratory distress, unconscious patients, acute chest pain, respiratory distress
- medium-acuity–expedient response, obeying traffic road rules within 30 minutes e.g. abdominal pain, many musculoskeletal injuries
- low-acuity–expedient response, obeying traffic road rules within 60 minutes e.g. generally unwell, vomiting with no other significant symptoms, chronic musculoskeletal problems

## Procedures

The analysis occurred in three stages. First, all the Referral Service cases referred for emergency ambulance dispatch were compared to the state-wide rates for paramedic treatment, and the metropolitan rates of paramedic transportation to hospital. (A metropolitan-only rate of paramedic treatment was not available.) Then, to provide more granularity, the appropriateness indicators were analysed according to dispatch acuity level and the most common presenting problems. Finally, the cases not transported following ambulance attendance underwent further descriptive analysis.

### Statistical analysis

Data were analysed using descriptive statistics, chi-squared tests of association, independent samples t-tests and logistic regressions to identify relationships with 95% CIs. All tests were considered to be significant at p<0.05. All statistical analysis was conducted using SPSS Version 23[19].

### Ethical approval

Ethical approval: Monash University Human Research Ethics Committee (MUHREC) (CF12/0547–2012000215). Organisational approval: Ambulance Victoria Research Governance Committee (R11-021).

## Results

**Fig 2:**
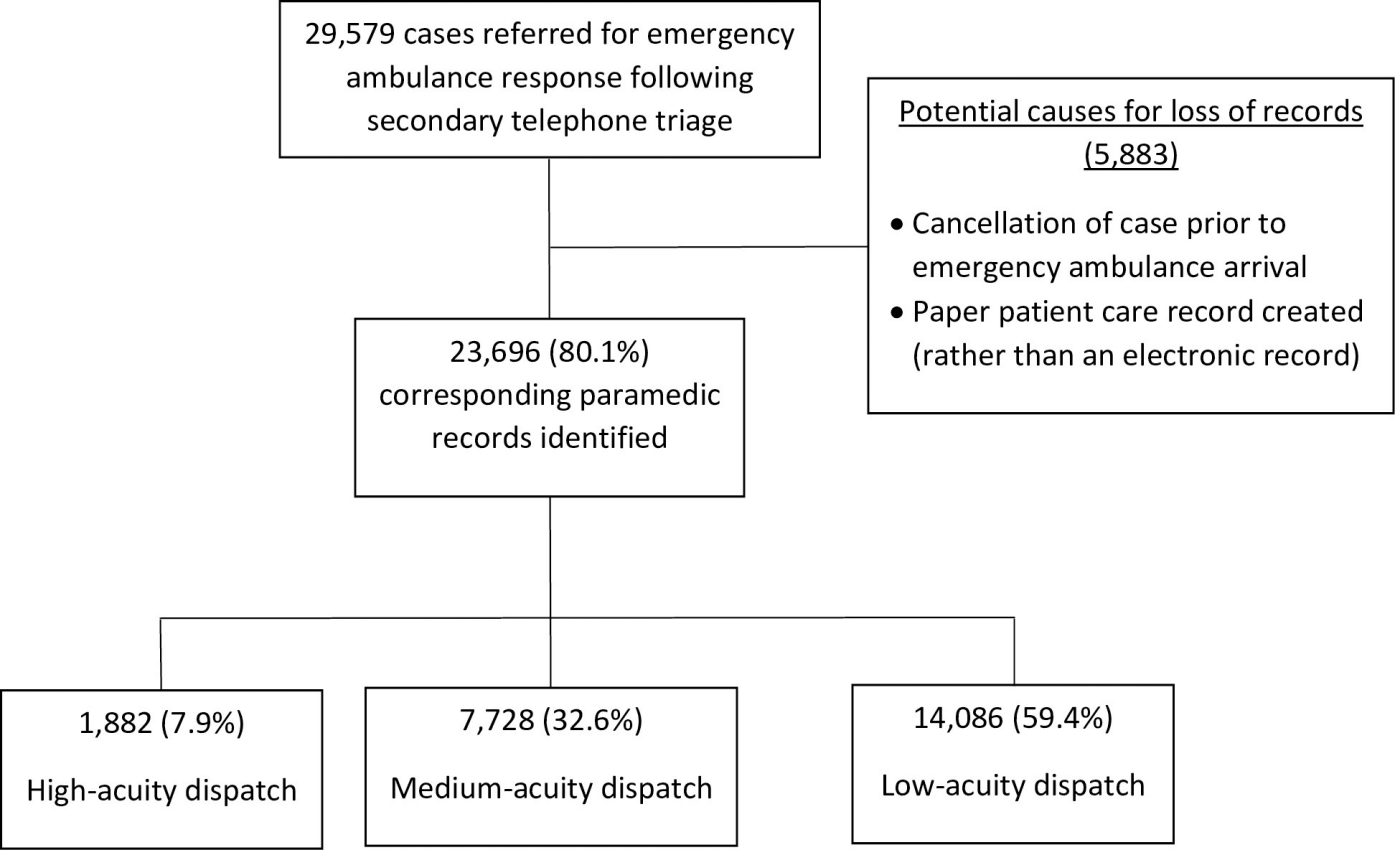
Case selection flow-chart.

During the study period 29,579 cases (27.6%) triaged by the Referral Service were referred for emergency ambulance dispatch and 23,696 (80.1%) were included in this study (Fig 2). Missing cases were cancelled prior to emergency ambulance arrival or had a paper patient care record created and were therefore not listed in the database (Fig 2).

The overall paramedic treatment rate for secondary telephone triage cases referred for emergency ambulance dispatch was similar to that of state-wide emergency ambulance cases (54.0% versus 55.5%). However, they demonstrated a significantly higher transportation rate (82.2%) than all emergency ambulance cases in Melbourne (71.1%) (Table 1). Two-thirds of the cases that were transported were also treated by paramedics (66.5%; n = 12,958).

**Table 1:** Ambulance outcomes for cases referred to emergency ambulance dispatch by the referral service compared to (1) the low-acuity emergency ambulance dispatch pathway, and (2) the ambulance outcomes for all emergency ambulance cases.

	Patient demographics		Paramedic treatment			Paramedic transport		
	Age (mean years)	Gender (% female)	%	Compared to cases referred as low-acuity	Compared to state-wide ambulance cases	%	Compared to cases referred as low-acuity	Compared to all metropolitan ambulance cases
**All emergency ambulance cases following RS triage**	57.6	54.9	54.0		OR 0.99 (95% CI 0.97–1.02; p = 0.49)	82.2		OR 1.76 (95% CI 1.7–1.8; p<0.001)
**High-acuity** (compared to low-acuity)	57.9	51.7	58.7	OR 1.25 (95% CI 1.13–1.38; p<0.001)	OR 1.22 (95% CI 1.11–1.34; p<0.001)	85.4	OR 1.3 (95% CI 1.13–1.48; p<0.001)	OR 2.23 (95% CI 1.96–2.5; p<0.001)
**Medium-acuity** (compared to low-acuity)	56.0	53.6	53.2	OR 0.988 (95% CI 0.935–1.05; p = 0.675)	OR 0.97 (95% CI 0.92–1.01; p = 0.132)	82.0	OR 1.0 (95% CI 0.93–1.08; p = 0.991)	OR 1.7 (95% CI 1.63–1.83; p<0.001)
**Low-acuity**	58.5	56.0	53.8		OR 0.98 (95% CI 0.95–1.01; p = 0.185)	82.0		OR 1.7 (95% CI 1.65–1.8; p<0.001)
**General ambulance population**			55.5 (State-wide)			71.1 (metropolitan)		

Of the cases referred for emergency ambulance dispatch, 7.9% (n = 1882) were as high-acuity, 32.6% (n = 7,728) as medium-acuity and 59.4% (n = 14,086) as low-acuity responses. The high-acuity dispatch cases had significantly higher rates of paramedic treatment and transport (Table 1) than both the low-acuity cases and the state-wide (treatment) or metropolitan (transport) ambulance cases. Finally, they were 9.5 times more likely to be transported using lights and sirens than low-acuity cases (95% CI 7.2–12.5; p<0.001). There was no statistically significant difference in provision of treatment or transport between those triaged to medium and low-acuity dispatch (Table 1). When the medium and low-acuity dispatch cases were compared to the state-wide (treatment) or metropolitan (transport) ambulance cases, no difference was found for paramedic treatment rates, however more patients were transported.

Analgesia was the most common paramedic treatment, followed by oxygen therapy (Table 2). The high-acuity cases had the highest proportion of cases treated in most treatment categories except for analgesia which had the lowest treatment rates.

**Table 2:** Frequency of paramedic treatment for acuity levels.

	Proportion of acuity-level treated % (N)		
	High-acuity dispatch	Medium-acuity dispatch	Low-acuity dispatch
Oxygen therapy	33.0 (622)	18.0 (1,393)	10.7 (1,509)
Perfusion /cardiovascular management	1.6 (30)	1.7 (135)	0.9 (122)
Mental health	0.2 (3)	0.1 (11)	0.2 (22)
Analgesia	23.9 (449)	33.7 (2,603)	42.4 (5,972)
Antiemetics	9.6 (180)	7.4 (571)	6.1 (856)
Intubation drugs	0.2 (4)	0.03 (2)	0.007 (1)
Sedation drugs (not for intubation)	0.2 (4)	0.2 (14)	0.2 (23)
Cardiovascular drugs	11.5 (217)	2.1 (166)	0.6 (84)
Respiratory drugs	1.8 (33)	0.5 (35)	0.3 (45)
Fluid administration	9.6 (180)	7.3 (567)	6.2 (867)
Other drugs	2.0 (38)	0.8 (60)	0.4 (52)

The most common triage guidelines used were abdominal pain (15.3%; n = 3627), back pain (9.7%; n = 2309) and dizziness/vertigo (6.7%; n = 1576). Over three quarters of the back pain (78.3%; n = 1809), and abdominal pain (75.5%; n = 2738) cases were treated by paramedics and this was predominantly with analgesia (back pain: 76.2%; abdominal pain 71.2%). Paramedics treated 43.3% (n = 682) of dizziness/vertigo cases and oxygen administration was the most common treatment provided (25.6%).

### Cases not transported following ambulance attendance

Overall, 17.7% (n = 4209) of cases were not transported by ambulance to hospital following paramedic assessment and of these only 2.8% received paramedic treatment. Patients not transported had a mean age of 54.3 years and 56.9% were female. Nearly two-thirds (60.3%) had been referred for low-acuity ambulance dispatch, 33.1% were dispatched as medium-acuity and 6.5% as high-acuity. The most common triage guidelines for this cohort of patients were abdominal pain (10.0%; n = 420), dizziness/vertigo (9.0%; n = 380) and back pain (7.6%; n = 319).

## Discussion

This was the first large-scale study of the appropriateness cases referred for emergency ambulance dispatch following low-acuity secondary telephone triage. The cases referred for emergency ambulance dispatch demonstrated paramedic treatment rates almost the same as state-wide ambulance cases and transportation rates higher than all metropolitan ambulance cases. When using the Victorian paramedic treatment rate as a benchmark for appropriateness, the results from this study suggest that the Referral Service appropriately identified cases similar to the state-wide ambulance cases, unless they were identified as high-acuity, in which case they were more appropriate for an emergency ambulance dispatch. The higher transportation rates also suggest that referral for emergency ambulance dispatch was appropriate. Nonetheless, the paramedic treatment rates, and to a lesser extent the transportation rates, were consistent with primary triage research[3, 13–18, 20, 21], demonstrating considerable overtriage remains.

Primary triage systems used by ambulance services have been designed to dispatch emergency ambulance quickly and therefore lack the granularity required to appropriately assess individual cases.[2] Despite their tendency to be risk-averse and overtriage, this lack of granularity means that patients requiring urgent medical attention can occasionally be classified as low-acuity. Prior to the introduction of secondary telephone triage, these cases could therefore wait up to an hour while cases classified as moderate or high-acuity were dispatched the available ambulances. Whilst these secondary telephone triage systems were introduced to manage low-acuity demand, they have also emerged as a safety net for primary triage systems by appropriately identify cases requiring a more urgent response. Despite the use of healthcare professionals, the persistent overtriage suggests that more needs to be done to assist with identifying patients suitable for alternative care pathways, and in providing suitable alternative care pathways for patients to be triaged to. A recent study of the population used in this research identified characteristics such as triage guideline, age and pain were associated with paramedic treatment[2]. From this, the presence of pain and subsequent analgesia administration suggested a case was appropriate for emergency ambulance dispatch. However, many communities are supported by services such as home-visiting doctors and non-emergency ambulance services, both of which can often administer analgesia and the latter can provide transport[22–25]. Yet these healthcare services often operate in silos, without communication or referral of patients between services. Moreover, a lack of knowledge about the characteristics of populations using these services means any relationships between services that do exist, may be relatively ineffective in meeting the needs of the patients they service. The Referral Service has long standing relationships with services such as these, yet these results indicate that more can be done with these relationship. A better understanding of the patient needs and expansion of the service provision for problems such as uncontrolled pain in the out-of-hospital setting could greatly improve ongoing patient care and reduce the demand on ambulance and ED resources.

The building of ongoing relationships with community-based healthcare providers, such a general practitioners, must also be encouraged to improve long-term outcomes, particularly for those suffering with chronic conditions like chronic pain[26, 27]. Referral of patients to these services, or to home-visiting services that then report to the patient’s usual healthcare provider may provide more timely and appropriate care, particularly in the setting of chronic conditions where ongoing management plans need to be considered. It is therefore important for multidisciplinary approaches to care for these patients be considered and used where appropriate to improve patient outcomes.

This also demonstrates how the structure of local healthcare system can shape and inform definitions of appropriateness for a secondary telephone triage service. Whilst paramedic treatment and transport were used as measures of appropriateness in this study, and in others[2, 3, 13–18, 28], there are many non-clinical reasons that warrant an ambulance dispatch. Non-clinical indicators involving patient welfare and safety, and the responsibility of ambulance services to play a productive and supportive role in the larger healthcare system are situations where overtriage is acceptable[29]. Therefore, any work refining secondary telephone triage must allow for ‘clinical need’ system dispositions to be overridden to ensure that the right care, clinical or not, is provided to the right patients.

The inability to link all of the data records between the Referral Service and paramedic datasets may have biased the results. Healthcare systems vary in their operational structures and functions, therefore the triage outcomes of secondary telephone triage services working within these systems will differ. This is also true for the role of ambulance services and paramedics. Different societal and operational expectations and requirements will result in different management approaches. The definition of ALS treatment also varies depending upon time, geography and jurisdictional resources, therefore studies using an ALS treatment measure will produce different findings depending on the setting. Using paramedic treatment and transport to determine appropriateness does not consider the non-clinical skills paramedics utilize or the alternative reasons for defining cases as appropriate for emergency ambulance dispatch[30]. Finally, the identification of cases that are ‘inappropriate’ for emergency ambulances does not suggest they are unsuitable for ED presentation.

## Conclusion

Overall, the cases returned for emergency ambulance dispatch following secondary telephone triage were appropriate. These cases demonstrated paramedic treatment and transportation rates at least the same as all state-wide and metropolitan emergency ambulance cases. Despite this, the paramedic treatment rates indicate considerable overtriage that should be further investigated to optimize the efficacy of secondary telephone triage.
